# Vascular Invasion of the Dental Epithelium Is Essential for Ameloblasts

**DOI:** 10.1177/00220345251341850

**Published:** 2025-06-26

**Authors:** H. Asrar, J.M. Fons, A.S. Tucker

**Affiliations:** 1 Centre for Craniofacial and Regenerative Biology, Faculty of Dentistry, Oral and Craniofacial Sciences, King’s College London, London, UK; 2 Institute of Histology and Embryology, First Faculty of Medicine, Charles University in Prague, Czech Republic

**Keywords:** tooth development, vascularization, enamel, VEGF, Hedgehog, Endothelial cells

## Abstract

The tooth is a highly vascularized organ. During odontogenesis, blood vessels enter the forming tooth through the dental papilla and surround the dental epithelium from the cap stage. We show that during the late bell stage, endothelial cells invade the outer enamel epithelium (OEE) and migrate through the stellate reticulum to vascularize the forming ameloblast layer. This migration was evident in both mouse and human tooth germs and is likely to represent a conserved mechanism. Migration was coordinated by dynamic changes in *Vegf* expression in the dental epithelium, with expression at the OEE at the bell stage shifting toward the ameloblast layer. Invasion through the OEE involved loss of integrity of the basement membrane, downregulation of tight junctions, and apoptosis of some OEE cells. Changes in the OEE were dependent on Hedgehog signaling, with a failure to invade in *K14creSmoothenedfl/fl* mice, where epithelial cells cannot respond to Hedgehog signaling. The impact of failed migration through the OEE was followed in *Cdh5creERT2 Vegfr2^fl/fl^* mutant mice, where the endothelial cells cannot respond to vascular endothelial growth factor (VEGF). Failure of OEE invasion resulted in differentiation defects and extensive cell death of the ameloblast layer, highlighting the essential requirement for vascularization for development of this layer. Our results reveal the essential role of Hedgehog and VEGF signaling in correct vascularization of the tooth germ epithelial layers, allowing breakdown of the OEE and targeting endothelial cell migration into the epithelium. A better understanding of the molecular regulation of endothelial cells will help decipher how this cell population interacts with different cells of the enamel organ and will aid in attempts to revascularize teeth.

## Introduction

The adult tooth has an extensive network of vasculature in the pulp that connects to the odontoblast layer. During development, the vasculature is laid down by endothelial cells that coalesce to form primitive vessels that undergo extensive remodeling to develop mature vessels (Schmeisser and Strasser 2002). During early odontogenesis, 2 distinct vascular networks have been identified: the dental papillary and the dental follicular vascular networks (Cerri et al. 2004). Mesoderm-derived endothelial cells wrap around the outer dental epithelium and move into the dental papilla during the late cap stage (Rothová et al. 2011). Textbooks have previously stated that the vasculature does not cross the outer enamel epithelium (OEE) to reach the ameloblast layer but rather that the stellate reticulum involutes to allow the vasculature to come in close contact with the ameloblast layer (Berkovitz et al. 2017). However, between the late cap to bell stage, capillaries have been shown to colonize the murine stellate reticulum, with an anastomosing capillary network reported around the stratum intermedium cells resting on top of ameloblasts postnatally in the mouse and rat (Cerri et al. 2004; Shadad et al. 2019). Involution of the stellate reticulum, therefore, appears to occur after blood vessel invasion of the vasculature, rather than instead of invasion (Baratella et al. 1999). Migration of the vasculature in the body is driven by vascular endothelial growth factor (VEGF), an essential chemoattractant orchestrating vascular assembly during organogenesis (Duffy et al. 2013). Genetic ablation of *Vegf* causes embryonic lethality due to circulatory/cardiac malformations as early as E8.5 (Shibuya 2011). The spatiotemporal expression pattern of *Vegf* mRNA has been analyzed during both embryonic and postnatal mouse development (Shadad et al. 2019). *Vegf* transcripts were localized in the primary enamel knot and underlying condensed mesenchyme during the early cap stage (Shadad et al. 2019). Between the late cap and late bell stage, *Vegf* activity was observed in all epithelial derivatives of the enamel organ (Shadad et al. 2019).

To colonize the epithelial layers, the endothelial cells need to migrate through the OEE. Changes in the OEE during the bell stage have been shown to involve Hedgehog signaling, with *K14creSmoothenedfl/fl* mutant mice having defects in polarization of the OEE, along with other defects such as fusion of the first and second molars (Gritli-Linde et al. 2002). In keeping with a role for Hedgehog in the dental epithelium, *Ptc1*, the receptor for Hedgehog signaling, was highly expressed along the molar OEE at P1 (Gritli-Linde et al. 2002)

Previous research suggested that capillaries were responsible for the transportation of calcium and phosphate to aid in the mineralization of enamel (Bernick 1960; Sasaki et al. 1984). In a similar function, the dental pulp vasculature has been shown to deliver the phosphate required for dentinogenesis (Matsubara et al. 2022).

In this study, we mapped the vasculature, using CD31 and CD34 as markers of endothelial cells, focusing on vascular invasion through the OEE to the SR and the relationship to forming ameloblasts. Vascularization patterns were compared at the bell stage in both mouse and human embryos to reveal conserved features. The roles of Hedgehog and VEGF signaling were assessed using mouse mutants, allowing an investigation of the role of the vasculature during development of the dental epithelium. Overall, our findings indicate that changes in the OEE allow endothelial cells to break through the epithelial barrier and invade the SR prior to any involution of this tissue during the bell stage of odontogenesis. This process was prevented in mice with loss of Hedgehog signaling in the epithelium in *K14creSmoothenedfl/fl* mutants and after disruption of *Vegf* signaling in *Cdh5creERT2Vegfr2flfl* mutants. Failure of vascular invasion led to a breakdown of the epithelial compartment of the enamel organ with a dramatic loss of the ameloblast layer, highlighting the essential early role for the vasculature in ameloblast development.

## Materials and Methods

### Samples

All mice were kept at a monitored temperature and relative air humidity with a 12-h light/dark cycle in pathogen-free barrier facilities at the New Hunts House biological services unit with food and water ad libitum. *K14cre* male mice were mated to *Smoothenedfl/fl* and *fl/+* females to generate *K14creSmoothened fl/+* males for mating to *Smoothenedfl/fl* females. *K14cre Smoothened fl/fl* mice were taken as embryos or at P1 (prior to the onset of any overt issues). *Cdh5creERT2* male mice were mated to *Vegfr2fl/fl* and *fl/+* females to generate *Cdh5 creERT2Vegfr2fl/+* males for mating to *Vegfr2fl/fl* females. All mice were maintained on a C57/Bl6 background. *Cdh5cre ERT2Vegfr2fl/fl* and *Vegfr2fl/fl* pups (controls) were injected intraperitoneally with tamoxifen (0.15 mg/g body weight) at P6 and P9 and culled at P10 or injected at P8 and culled at P12. Pregnant mice, embryos, and neonates were culled using approved schedule 1 culling methods. Mutant and control mice were age matched, with both male and female samples analyzed.

Mouse research was approved by the Animal Care and Ethics Committee of Kings College London, following a study plan, and was covered by UK Home Office Institution, project and personal licenses. Animal work followed the Animal Research: Reporting of In Vivo Experiments 2.0 (ARRIVE 2.0) guidelines.

Human embryos/fetuses were provided by the Human Developmental Biology Resource (HDBR) with ethical approval provided by Newcastle University (Project 200665).

See the appendix for additional methods.

## Results

### Blood Vessels Migrate through the OEE at the Bell Stage of Tooth Development

**Figure 1.**
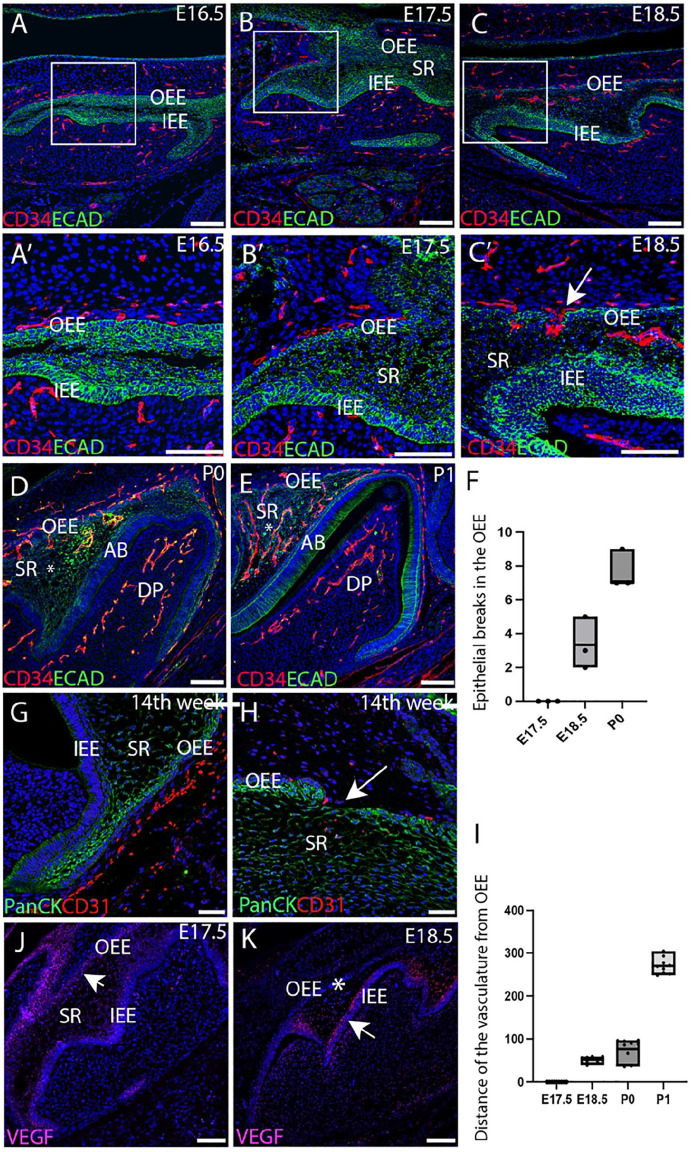
Blood vessel migration through the outer enamel epithelium at the bell stage of tooth development. (**A–E, J, K**) Mouse embryonic development. (**A–E**) Immunofluorescence CD34 (endothelial marker) in red. E-cadherin (epithelial marker) in green. (**J, K**) *Vegf* RNAscope. (**A, A′**) E (embryonic day) 16.5, (**B, B′**) E17.5, (**C, C′**), E18.5. (**C′**) Break in the outer enamel epithelium highlighted by the white arrow. (**D**) P (postnatal day) 0, (**E**) P (postnatal day) 1. The asterisk in D and E indicates the stellate reticulum (SR). (**G, H**) Human deciduous molar tooth development at 14 wk after conception. Immunofluorescence CD31 (endothelial marker) in red. Pan-cytokeratin (epithelial marker) in green. (**H**) Break in the outer enamel epithelium highlighted by the white arrow. (**J**) E17.5. The arrow in J indicates high *Vegf* expression at the outer enamel epithelium. (**K**) E18.5. The arrow in K indicates high *Vegf* expression shifted to near the inner enamel epithelium. The asterisk in K highlights the absence of *Vegf* in the outer enamel epithelium by E18.5. (**F**) Quantification of the number of epithelial breaks (breaches of the outer enamel epithelium by endothelial cells). *N* = 3 mice (average of 3 sections per mouse). (**I**) Quantification of the distance in µm of the vasculature from the outer enamel epithelium (OEE). The furthest endothelial cells from the OEE were measured for each section (see Appendix Fig. 3). *N* = 3 mice (average of 3 sections per mouse). AB, ameloblasts; DP, dental papilla; IEE, inner enamel epithelium; OEE, outer enamel epithelium; SR, stellate reticulum. Scale bars = 100 µm.

To explore the relationship between the dental epithelium and the surrounding vasculature during differentiation, endothelial cells were investigated in the first molar (M1) from the early bell stage. In the mouse, CD34, a marker for endothelial precursor cells called hemangioblasts (Nagy-bota et al. 2014), was compared with E-cad, which labels the dental epithelium. At E16.5 and E17.5, the intricate network of CD34+ve blood vessels was closely associated with the OEE but did not penetrate into the epithelial layers (Fig. 1A, A′, B, B′). By E18.5, interruptions in the OEE were evident, with invasion of the CD34+ve endothelial cells through the OEE into the stellate reticulum (Fig. 1C, C′, Appendix Fig. 3). Blood vessels continued to invade the stellate reticulum during the early postnatal stages through breaks in the OEE (Fig. 1D, E). Quantification of the number of breaks observed in a section revealed an increase in the number of breaks between E18.5-P0, enabling vascular migration into the stellate reticulum (Fig. 1F). By P1, abundant CD34+ve cells had colonized the stellate reticulum, residing close to the stratum intermedium and the newly differentiated ameloblast layer at a distance from the OEE (Fig. 1E, I). The influx of endothelial cells correlated with waves of amelogenin expression, which started at the tips of the cusps at E18.5 before progressing more apically, suggesting links between enamel differentiation and vascularization (Appendix Fig. 4). A similar invasion of the vasculature through the OEE was conserved during human deciduous molar tooth development at 14 wk of gestation. CD31+ve cells were observed lined up along the pancytokeratin+ve OEE with migration of endothelial cells through breaks in the integrity of the epithelium (Fig. 1G, H). Complementary vascular migratory patterns through the OEE were therefore identified in both mammalian species during the bell stage of tooth development.

The key angiogenic signal that orchestrates vascular formation during tooth development is VEGF. The expression of *Vegf* was therefore followed during migration of the vasculature. At E17.5, strong expression of *Vegf* was associated with the OEE, with weaker expression in the stellate reticulum (Fig. 1J). By E18.5, *Vegf* expression had shifted to high levels in the stratum intermedium above the developing ameloblast layer (Fig. 1K). The expression of *Vegf*, therefore, mimicked the changing pattern of vasculature and was likely to be the driving force in attracting the endothelial cells through the stellate reticulum to the ameloblast layer.

### Laminin Breakdown and Sporadic Apoptosis Contribute to Vascular Invasion through the OEE

**Figure 2.**
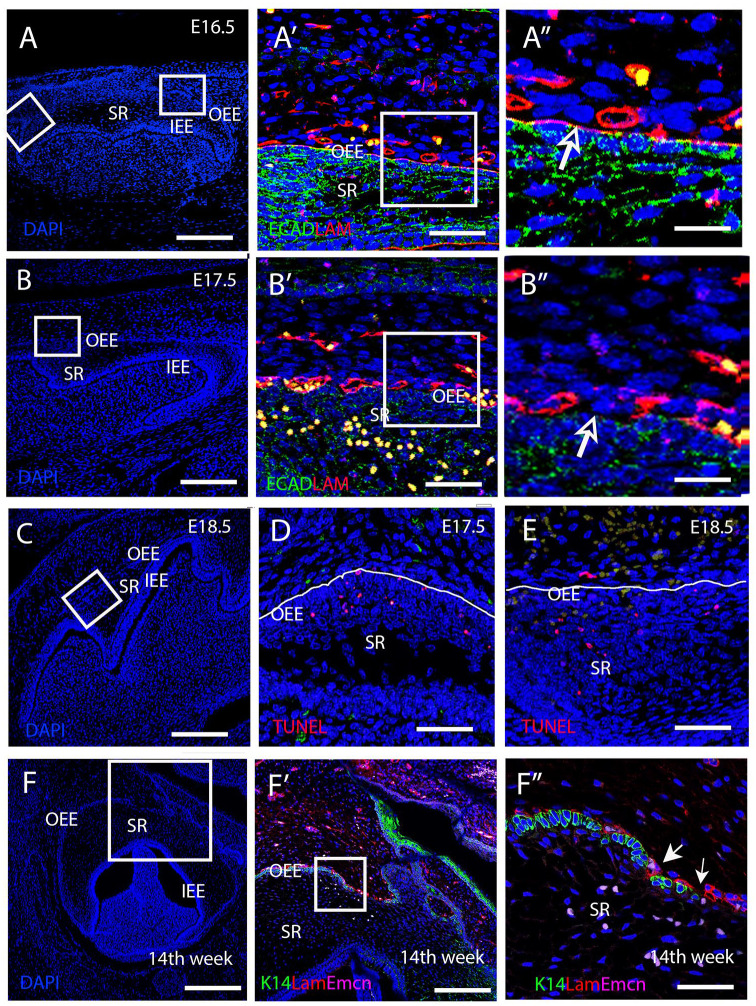
Loss of laminin and apoptosis facilitate vascular invasion into the stellate reticulum. (**A–E**) Mouse embryo. (**A-A**″) E16.5. (**B-B**″, **D**) E17.5. (**C, E**) E18.5. (**A, B, C, F**) DAPI images of tooth germs to show the selected regions of interest at each stage. Boxed areas in A,B shown in A′, B′. (**A**′, **A**′′, **B**′, **B**′′) Immunofluorescence laminin (Lam), E-cadherin (ECAD) (green). Boxed areas in A′,B′ shown in A″, B″. Arrows in A″, B″ show the presence and lack of laminin in the basement membrane. (**D, E**) TUNEL staining (red). (**D**) E17.5, equivalent region to that shown in the boxed area in B. (**E**) E18.5, equivalent region to that shown in the boxed area in C. Some positive apoptotic cells are associated with the OEE at both stages. The white line outlines the edge of the OEE. (**F–F**″) Human deciduous molar tooth 14 wk after conception. (**F**′**–F**″) Immunofluorescence of Keratin14 (K14) (green). Endomucin (Emcn) (magenta). Boxed areas in F shown in F′. Breaks in the OEE are associated with the loss of laminin and downregulation of Keratin14. The boxed areas in F′ shown in F″. The arrow in F″ highlights the break point with endomucin-positive endothelial cells entering the epithelium. IEE, inner enamel epithelium; OEE, outer enamel epithelium; SR, stellate reticulum. Scale bar in A, B, C, F′ = 100 µm; A′, B′, D, E = 40 µm; A′, B″ = 20 µm; F = 250 µm; F″ = 25 µm.

To understand the underlying mechanism responsible for vascular migration through the OEE, changes in the basement membrane were investigated using laminin as a marker. At E16.5 in the mouse, a uniform layer of laminin outlining the basement membrane of the OEE was observed, in addition to expression around forming blood vessels (Fig. 2A–A″). However, by E17.5, disruptions in laminin expression around the OEE were evident (Fig. 2B–B″). A similar interruption of the basement membrane was evident at 14 wk of gestation during human molar development (Fig. 2F–F″). Breaks in the integrity of the OEE, as shown by lack of Keratin14 expression, coincided with endothelial invasion into the stellate reticulum, as revealed using endomucin, a blood vessel marker (Fig. 2F′–F″). To assess if apoptosis was linked to the disruption of the epithelial barrier allowing vascular migration, TUNEL staining was conducted. A few TUNEL+ve cells were located in the OEE at E17.5 prior to invasion of the vasculature (Fig. 2D). In addition, some TUNEL+ve cells were evident in the stellate reticulum at E17.5 and E18.5 (Fig. 2C, D, E). Our findings suggest that apoptosis and basement membrane disintegration may provide a mechanism to facilitate vascular migration.

### Vasculature Fails to Invade the Stellate Reticulum due to a Loss of Hedgehog Signaling

**Figure 3.**
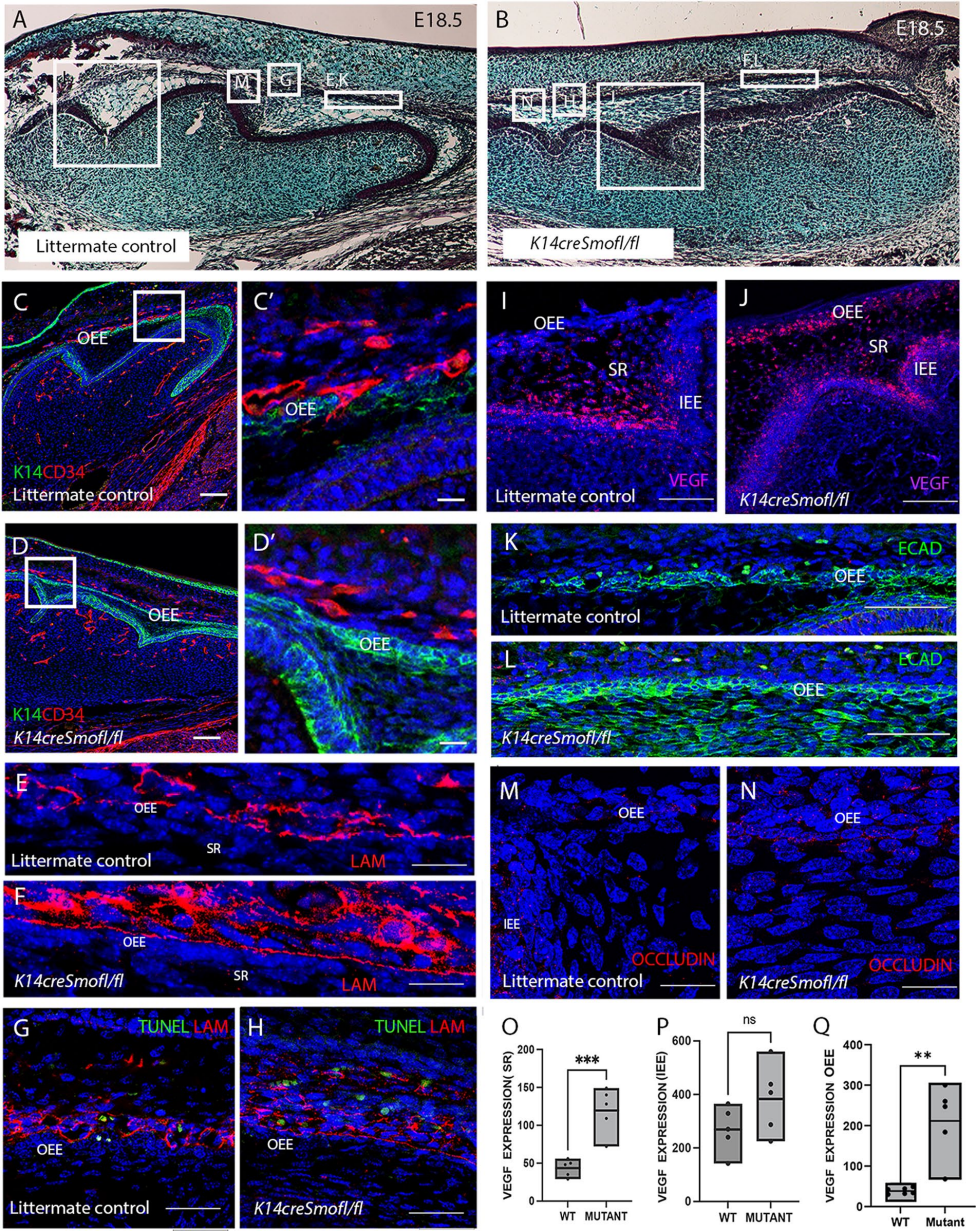
Endothelial cells fail to migrate into the SR due to loss of Hedgehog signaling. (**A–H**) Mouse embryos E18.5. Samples collected in the morning. (**A, C, C′, E, G, I, K, M**) *Smoothenedfl/fl* littermate controls. (**B, D, D′, F, H, J, L, N**) *K14creSmoothenedfl/fl* embryos. (**A, B**) Histology stain. Boxed areas highlight the representative areas shown in immunofluorescence images. (**C, C′, D, D′**) Immunofluorescence Keratin14 (KRT14) green, CD34 (red). The boxed area in C and D, shown in C′, D′. (**C, C**′) Endothelial cells have started to invade the OEE. **(D, D**′) Endothelial cells remain outside the OEE. (**E**) *Smoothenedfl/fl* embryo with loss of laminin in the basement membrane along the outer enamel epithelium. (**F**) *K14creSmoothenedfl/fl* embryo with robust laminin staining retained along the outer enamel epithelium. (**G, H**) TUNEL stain (green). Laminin (red). Apoptotic cells were evident around the OEE in both mutant and controls. In controls (**G**), TUNEL cells were associated with breaks in laminin expression. (**I, J**) *Vegf* RNAscope. (**I**) *Smoothenedfl/fl* embryo with *Vegf* expression focused at the IEE and absent from the OEE. (**J**) *K14creSmoothenedfl/fl* embryo with *Vegf* expression focused at the OEE, SR, and IEE. (**K, L**) Immunofluorescence E-cadherin (green). (**K**) *Smoothenedfl/fl* embryo with a break in Ecad at the OEE. (**L**) *K14creSmoothenedfl/fl* embryo with maintained Ecad at the OEE. (**M, N**) Immunofluorescence occludin. (**M**) *Smoothenedfl/fl* embryo with downregulation of occludin at the OEE. (**N**) *K14creSmoothenedfl/fl* embryo with maintained occludin at the OEE. (**O–Q**) Quantification of *Vegf* levels by RNAscope in mutant and control mice (*N* = 5). (**O, Q**) Significant differences in vascular endothelial growth factor (VEGF) in the SR (**O**) (*P* = 0.0008) and OEE (**Q**) (*P* = 0.008) but not at the (**P**) IEE (*P* = 0.14). IEE, inner enamel epithelium; OEE, outer enamel epithelium; SR, stellate reticulum. Scales bars C, D = 50 µm. Scale bars C′, D′, E, F, M, N = 10 µm; G, H, K, L = 20 µm; I, J = 100 µm.

Sonic Hedgehog has been shown to regulate the growth, polarization, and proliferation of the dental epithelium (Gritl-Linde et al. 2002). Lack of Hedgehog signaling led to a fused molar phenotype with disruptive changes in the inner enamel epithelium (Gritl-Linde et al. 2002) (Fig. 3A, B). To investigate if Hedgehog signaling participates in vascular migration through the OEE, we analyzed *K14creSmoothenedfl/fl* mutant mice. Vascular analysis revealed normal blood vessel migratory patterns in Cre-negative *Smoothened fl/fl* controls, with endothelial cells pushing through the OEE into the stellate reticulum at E18.5 (Fig. 3C–C′). In contrast, the mutants showed a failure of CD34+ve cells to invade through the OEE (Fig. 3D–D′). To understand the reason for the lack of invasion, the integrity of the basement membrane was assessed by laminin expression. In the control, laminin expression was disrupted along the OEE, while in *K14creSmoothenedfl/fl* mutants, the basement membrane remained intact with strong expression of laminin (Fig. 3E, F). In the control samples, TUNEL-positive apoptotic cells were evident close to the OEE, corresponding to breaks in the basement membrane (Fig. 3G). In the mutants, TUNEL-positive cells were also located near the OEE cells but did not correspond to loss of laminin (Fig. 3H). This suggests that apoptosis may not be sufficient to cause a break in the OEE.

The failure of blood vessel migration could be due to loss of VEGF as an attractant. In the control, *Vegf* mRNA expression was restricted to the stellate reticulum and stratum intermedium, close to the inner enamel epithelium at E18.5 (Fig. 3I). Interestingly, in the mutants, all epithelial derivatives showed high levels of *Vegf* expression (Fig. 3J). Quantification revealed significant upregulation of *Vegf* expression (as calculated by RNAscope) at the OEE and stellate reticulum of the mutant group relative to the control group (Fig. 3O, Q), with a nonsignificant change at the inner enamel epithelium (Fig. 3P). These results suggest a feedback mechanism that caused increased *Vegf* expression in the stellate reticulum as a consequence of the lack of vascular invasion in the mutant.

To explore the cellular changes in the outer enamel epithelial cells of the *K14creSmoothenedfl/fl* mutant, a tight junction marker occludin was analyzed. In littermate controls, the OEE showed gaps in E-cadherin associated with overlying autofluorescent blood cells (Fig. 3K) and low levels of occludin expression (Fig. 3M). In contrast, E-cadherin expression in the mutant OEE was maintained with no disruption (Fig. 3L) and high levels of occludin expression (Fig. 3N).

### Breakdown of the Stellate Reticulum and Loss of Ameloblasts due to Lack of Vascular Invasion

**Figure 4.**
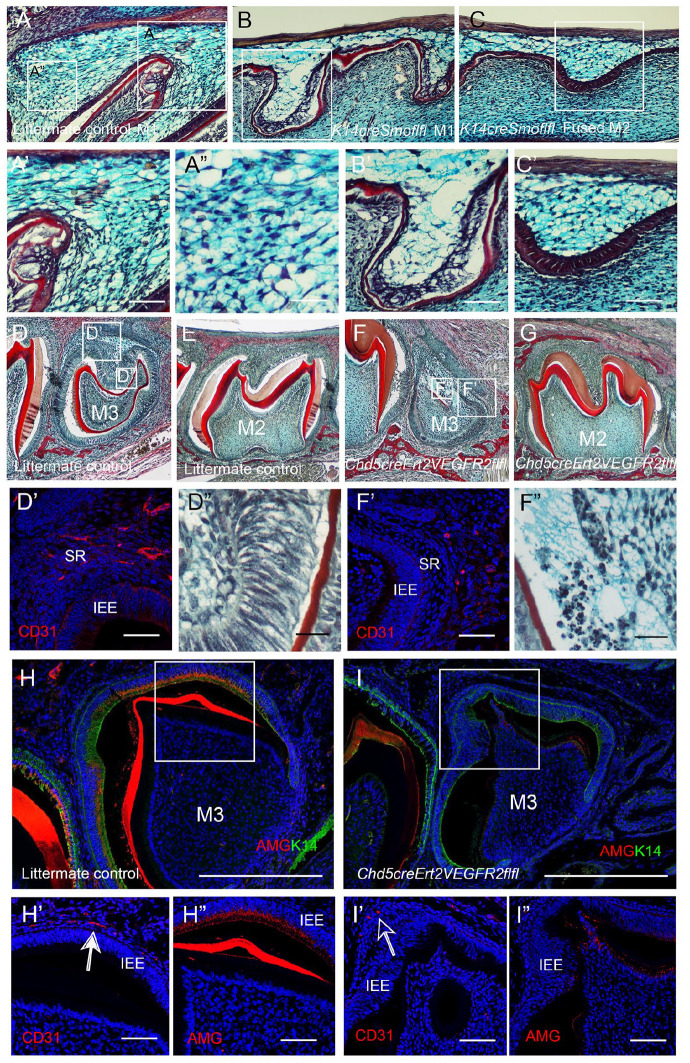
Failure of endothelial cell migration through the OEE causes disruption of the SR and loss of the ameloblast layer. (**A–C**′) Postnatal day 1 (P1) mouse. (A–C′, D–G, D″, F″) Trichrome histology stain. (A, A′, A″) *Smoothenedfl/fl* pup. Boxes in A indicate highlighted region in A′, A″. (A′, A″) Blood cells can be observed in the intact stellate reticulum of the first molar (M1). (**B, C**) *K14creSmoothenedfl/fl* pup. (B) First molar (M1). Box in B indicates the highlighted region in B′. (B′) Large holes are evident in the SR. (C) Second molar (M2), fused to M1 in the mutant. Box in C indicates the highlighted region in C′. (C′) The later-developing M2 still has an intact SR layer. (D, D′, D″, E) *Vegfr2fl/fl* pups P10. (F, F′, F″, G) *Cdh5creERT2Vegfr2fl/fl* pups P10. (**D, F**) Third molar M3. (**E, G**) Second molar M2. Boxes in D and F indicate highlighted regions in D′, D″, F′, F″. Tamoxifen injected at P6 and P9. (E, G) The gross morphology of the second molar (M2) was unaffected in the mutant. Dentin staining in red. (D′, F′) Immunofluorescence CD31 (red). Endothelial cells are found within the stellate reticulum (SR) of the third molar (M3) by P10 in the control (D′) but not the mutant (F′). (D″, F″) The morphology of the M3 was severely disrupted with loss of the ameloblast layer. Dentin staining in red. (D″) *Vegfr2fl/fl* pup with columnar ameloblasts. (F″) *Cdh5creERT2Vegfr2fl/fl* pup with dark condensed apoptotic bodies where the ameloblasts should have been. (H, H′, H″) *Vegfr2fl/fl* pup P12. (I, I′, I″) *Cdh5creERT2Vegfr2fl/fl* pups P12. Tamoxifen injection P8. (**H, I**) Immunofluorescence amelogenin (red), Keratin14 (green). Boxes in H,I highlight regions in H′, H″, I′, I″. (H′, I′) Immunofluorescence CD31 (red). The arrow in I′ points to the vasculature right up against the ameloblasts in the IEE. (H″, I″) Immunofluorescence amelogenin (red). Red in dentin is autofluorescence. The control ameloblasts express amelogenin, which is absent in the mutants. IEE, inner enamel epithelium; M1, first molar; M2, second molar; M3, third molar; OEE, outer enamel epithelium; SR, stellate reticulum. Scale bars A′, B′, C′, D′, F′, H′H″, I′,I″ = 100 µm. Scale bar A″, D″, F″ = 50 µm.

To assess whether failure of the vasculature to invade the epithelium had any consequence on the developing enamel organ, samples were collected at P1. The *K14creSmoothenedfl/fl* mutants die perinatally, so this represents the oldest stage available for analysis. Trichrome analysis of the first molar confirmed a densely populated stellate reticulum invaded by the vasculature in the littermate controls (Fig. 4A-A′′). In contrast, few widely dispersed stellate reticulum cells were observed near the mesial end of the fused M1 region in the Hh mutant (Fig. 4B,B′). Notably, numerous stellate reticulum cells were present in the distal end of the fused M2 of the *K14creSmoothenedfl/fl* mutant (Fig. 4C, C′). The presence of an intact densely populated SR in the M2 region of the tooth, in contrast to the developmentally more mature M1 region, indicated that disintegration of the SR was a direct consequence of the failure of vascular invasion. The *K14creSmoothenedfl/fl* mutants additionally have defects in development of the inner enamel epithelium, which are likely to be a direct effect of a loss of Shh signalling in these tissues (Gritli-Linde et al. 2002).

To differentiate between the impact of the loss of Hh signaling on the epithelium from the failure of blood vessel invasion, we moved to an endothelial-specific mouse model. The *Cdh5creERT2 Vegfr2^fl/fl^* mutant genetically ablates *Vegfr2* from endothelial cells, removing the ability of endothelial cells to respond to VEGF signaling and thereby resulting in the failure of vascular migration. Since conditional *Vegfr2* deletion in endothelial cells at embryonic stages is lethal, samples were analyzed at postnatal stages with a focus on the third molar (M3), which reaches the bell stage postnatally (Chlastakova et al. 2011). To target the M3, tamoxifen was injected at P6 and P9, or P8. At this time point, the vasculature has already moved into the dental papilla to reach the odontoblast layer, so this method allowed for targeting of the later migration through the epithelium. By P10, the control M3 had reached the bell stage with deposition of dentine, while the mutant M3 was severely disrupted (Fig. 4D, F). In contrast, vascularization of the epithelium of the M2 would have already occurred before the application of tamoxifen, so the M2 would not be predicted to be affected greatly by the postnatal window of injection. In keeping with this, the mutant M2 appeared overtly normal morphologically at P10 (Fig. 4E, G). In control littermates at P10, CD31+ve cells had invaded the OEE of the M3 (Fig. 4D′). However, endothelial cells failed to migrate into the stellate reticulum of the mutant third molar by this stage (Fig. 4F′). The ameloblasts in the control showed a normal elongated appearance lining the dentin layer of the dental papilla (Fig. 4D″). In constrast, the mutant ameloblasts were lost, with evidence of cell death as seen by numerous apoptotic bodies (Fig. 4F″). By P12, the M3 ameloblast layer started to express amelogenin, with the vasculature closely associated with the IEE (Fig. 4H–H″). In contrast, no amelogenin was evident in the mutants, where the vasculature was at a distance from the IEE (Fig. 4I–I″). A failure of invasion of the vasculature, therefore, had profound consequences on the development of ameloblasts.

## Discussion

Previous studies have focused on following vascularization of the odontoblast layer and dental papilla during odontogenesis (Yuan et al. 2014; Shadad et al. 2019). In this article, we instead highlight the essential requirement for vascularization of the enamel organ during differentiation of the molar tooth germ. During the late bell stage in both human and mouse embryos, endothelial cells were observed to migrate through the OEE at the top of the tooth and to move through the stellate reticulum to the forming ameloblast layer. Vascularization of the epithelium, therefore, predates any involution and collapse of the stellate reticulum. Migration of the vasculature was driven by changing patterns of *Vegf* expression, which moved from the OEE to near the stratum intermedium.

We show that migration required changes in the OEE, with downregulation of tight junctions and loss of basement membrane integrity, which were dependent on Hedgehog signaling. Apoptosis also appeared to play a role, potentially to create gaps in the OEE; however, this process did not appear to be dependent on Hedgehog signaling. Sonic Hedgehog is expressed in the stratum intermedium during the bell stage, and it is likely that this layer provides a source of Hedgehog ligands to control changes in the OEE (Koyama et al. 2001). Interestingly, previous reports have shown that Hedgehog regulates E-cadherin expression in epithelial cells, with the loss of Sonic Hedgehog leading to the dissociation of E-cadherin and the ZO-1/occludin complex (Xiao et al. 2010). Similarly, endogenous Sonic Hedgehog regulated bovine retinal endothelial tight junction expression maintenance as inhibition with cyclopamine led to defective barrier properties (Díaz-Coránguez et al. 2017). These findings directly contradict our results, in which a lack of Hedgehog signaling during tooth development led to the persistence of ZO-1 and E-cad expression in the OEE. These differences suggest that the role of Hedgehog signaling in the maintenance of tight junctions is context dependent and could be a result of tissue, species, or stage variation.

**Figure 5.**
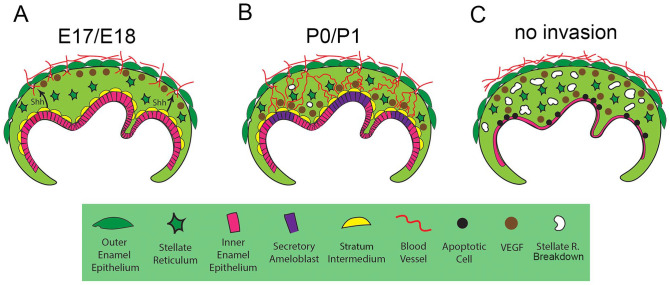
Schematic showing the signals involved in vascularization of the ameloblast layer. (**A**) By E17.5/18.5, Hedgehog signaling from the stratum intermedium (yellow cells) leads to changes in the permeability of the outer enamel epithelium. Endothelial cells (red) move in responding to high levels of *Vegf* (brown circles) expressed at the outer enamel epithelium. (**B**) By P0/P1, *Vegf* expression (brown circles) has shifted to near the inner enamel epithelium, with the endothelial cells (red) moving through the stellate reticulum (star shaped cells) to reach the ameloblast layer (pink/purple cells). (**C**) If the endothelial cells fail to enter through the outer enamel epithelium (due to maintained integrity of the outer enamel epithelium or due to an inability to respond to *Vegf*), the stellate reticulum and ameloblast layer become disorganized and undergo apoptosis.

In the absence of Hedgehog signaling in the epithelium, the blood vessels failed to migrate into the epithelial layers. Interestingly, this led to a compensatory upregulation of *Vegf*, with higher levels in the stellate reticulum, potentially in response to hypoxia in these tissues. In the *Smoothened* KO, the stellate reticulum layer started to thin out by P1, which could be a consequence of the direct loss of Hh signaling or due to the absence of blood vessel invasion. A similar disruption to the stellate reticulum, and the ameloblast layer in particular, was evident when blood vessels failed to enter the epithelium, due to conditional knockout of *Vegfr2* in endothelial cells. Loss of vasculature invasion had severe consequences for the inner enamel epithelium and the expression of amelogenin, highlighting the importance of a blood vessel supply for ameloblast differentiation and survival. Prevention of vascular invasion, either through disrupting migration cues or preventing access through the OEE, led to disruption of the enamel organ, highlighting the importance of coordinating migration cues with changes in the OEE (Fig. 5A–C). The ability to target the specific window when the invasion of the OEE occurs makes the *Vegfr2* conditional knockout particularly powerful and avoids direct effects on the ameloblast layer, as observed in Hedgehog mutants. Recently, a novel endothelial subtype was identified in the mesenchymal dental papilla that was specific to dentinogenesis (Matsubara et al. 2022). These cells were influenced by the odontoblasts and in turn triggered odontoblast maturation. A similar relationship, therefore, appears likely with respect to the endothelial cells that invade the epithelium of the tooth to reach the ameloblasts. For odontoblasts, Tgfb and pleiotrophine have been suggested to be produced by the vasculature (Matsubara et al. 2022). Future studies could therefore investigate whether ameloblasts share these signaling cues or are stimulated by a unique set.

Overall, this article highlights the signals required to coordinate vascularization of ameloblasts through the OEE (Hh and *Vegf*) and the essential nature of this route for normal tooth development.

## Author Contributions

H. Asrar, contributed to design, data analysis, drafted the manuscript; J.M. Fons, contributed to design, data analysis, critically revised the manuscript; A.S. Tucker, contributed to design, critically revised the manuscript. All authors gave final approval and agree to be accountable for all aspects of the work.

## Supplemental Material

sj-docx-1-jdr-10.1177_00220345251341850 – Supplemental material for Vascular Invasion of the Dental Epithelium Is Essential for Ameloblasts Supplemental material, sj-docx-1-jdr-10.1177_00220345251341850 for Vascular Invasion of the Dental Epithelium Is Essential for Ameloblasts by H. Asrar, J.M. Fons and A.S. Tucker in Journal of Dental Research
