# Supplementary material for: Vascular Invasion of the Dental Epithelium Is Essential for Ameloblasts
Source: J Dent Res. 2025 Jun 26;104(13):1547–55. doi: 10.1177/00220345251341850 (PMC12578956; doi:10.1177/00220345251341850)
Supplement: sj-docx-1-jdr-10.1177_00220345251341850 – Supplemental material for Vascular Invasion of the Dental Epithelium Is Essential for Ameloblasts [file sj-docx-1-jdr-10.1177_00220345251341850.docx]

*Appendix:*

*Methods:*

*Histology:*

Serial tissue sections of the molars were stained with trichrome (picrosirius red, alcian blue, and haematoxylin) to identify morphological features (Appendix Figure 1).

*Immunofluorescence and RNAscope:*

Immunofluorescence staining was conducted for CD34, CD31, Endomucin, Laminin, Occludin, Zo-1, Ecad, Keratin 14, and Amelogenin (Appendix Table 1A). After deparaffinization using Neoclear and rehydration in a declining series of ethanol dilutions, paraffin sections underwent antigen retrieval in Tris EDTA PH= 9 or citric acid. Sections were blocked in Blocking reagent and Goat Serum. Sections were then treated overnight at 4°C with primary antibodies diluted in blocking buffer in a moisturised chamber. Slides were washed and incubated with secondary antibodies (Appendix Table 1A,B) diluted in blocking buffer for 2 hours at RT in the dark. Nuclear counterstaining was performed using DAPI (Fluoroshield^TM^ Sigma-Aldrich). Negative control staining was carried out by omitting the primary antibody (Appendix Figure 2).

RNAscope from Advanced Cell Diagnostics (RNAscope® Multiplex Fluorescent Detection Reagents Kit v2) was used following the manufacturer's instructions. A *VegfA* probe was utilized (Mn-Vegf O 436961) at a 1:50 dilution. Slides were imaged on a confocal microscope (ZEISS LSM 980) and ZEISS Apotome.2. Experimental data was analysed and quantified using ImageJ.

*TUNEL:*

A TUNEL assay was performed using the *In situ* apoptosis detection kit (TaKaRa, cat. #MK500) following the manufacturer's instructions to reveal apoptotic cells.

*Quantification:*

For *Vegf* RNAscope, the number of positive dots was counted on a section from 5 mutants and 5 littermate controls, where N = a mouse. Dots at the IEE, SR and OEE were counted within a rectangle of set size, so that each area had the same number of cells. For each sample the same positions were used to ensure standardisation.

For quantification of the number of breaches of the outer enamel epithelium and the distance of the endothelial cells from the outer enamel epithelium N = 3 mice were counted with an average taken from 3 sections per mouse. For the distance measure, the endothelial cells furthest from the OEE was measured (see Appendix Figure 3). Measurements and counting were not blind.

*Statistical analysis:*

Power calculations were used to determine the sample size (minimum N=3), using a large effect size and 80% power. Statistical analyses were performed using GraphPad Prism 10 software (minimum N=5). The normality of the original data was assessed using a Kolmogorov Smirov Test of Normality for assessing Data Distribution, followed by a Levenes Test for Homogeneity of Variance. For normally distributed datasets an unpaired 2 tailed t-test was performed. Where the homogeneity of variance was not met a Mann-Whitney U test was performed. Data were expressed as the mean ± standard deviation. A statistically significant difference was defined as *p-*values <0.05. Animals were selected based on genotype.

Appendix Table 1A: Primary antibodies

| **Primary**  **Antibody** | **Antibody**  **Code** | **Antigen Retrieval** | **Proteinase K** | **Concentration** | **Blocking** | **Amplification** |
| --- | --- | --- | --- | --- | --- | --- |
| CD34 | Ab8158 | Citric Acid | No | 1:300 | 2 hours | No |
| CD31 | Ab28364 | Citric Acid | No | 1:300 | 2 hours | No |
| Endomucin | Ab106100 | Tris EDTA | Yes | 1:200 | 1 hour | No |
| Laminin | L9393 | Tris EDTA | No | 1:500 | 1 hour | No |
| Occludin | ab31721 | Citric Acid | No | 1:500 | 1 hour | No |
| ZO-1 | SC10804 | Tris EDTA | No | 1:200 | 2 hours | No |
| Ecad | Ab76055 | Citric Acid | No | 1:200 | 2 hours | No |
| Ecad | Ab76319 | Citric Acid | No | 1:200 | 2 hours | No |
| Keratin 14 | Ab7800 | Citric acid | No | 1:200 | 2 hours | No |
| Amelogenin | Ab153915 | Citric acid | No | 1: 200 | 2 hours | Yes |
| Pan-cytokeratin | Ab961 | Citric acid | No | Neat | 2 hours | No |

Appendix Table 1B: Secondary antibodies

| **Secondary Antibody** | **Manufacturer** | **Dilution** | **Host** |
| --- | --- | --- | --- |
| Alexa Fluor 568 | Invitrogen | 1:500 | Donkey |
| Alexa Fluor 488 | Invitrogen | 1:500 | Donkey |
| Alexa Fluor 647 | Invitrogen | 1:500 | Donkey |
| Anti-Rabbit Biotinylated | Abcam | 1:500 | Goat |

Appendix Figure 1: Histology tooth development


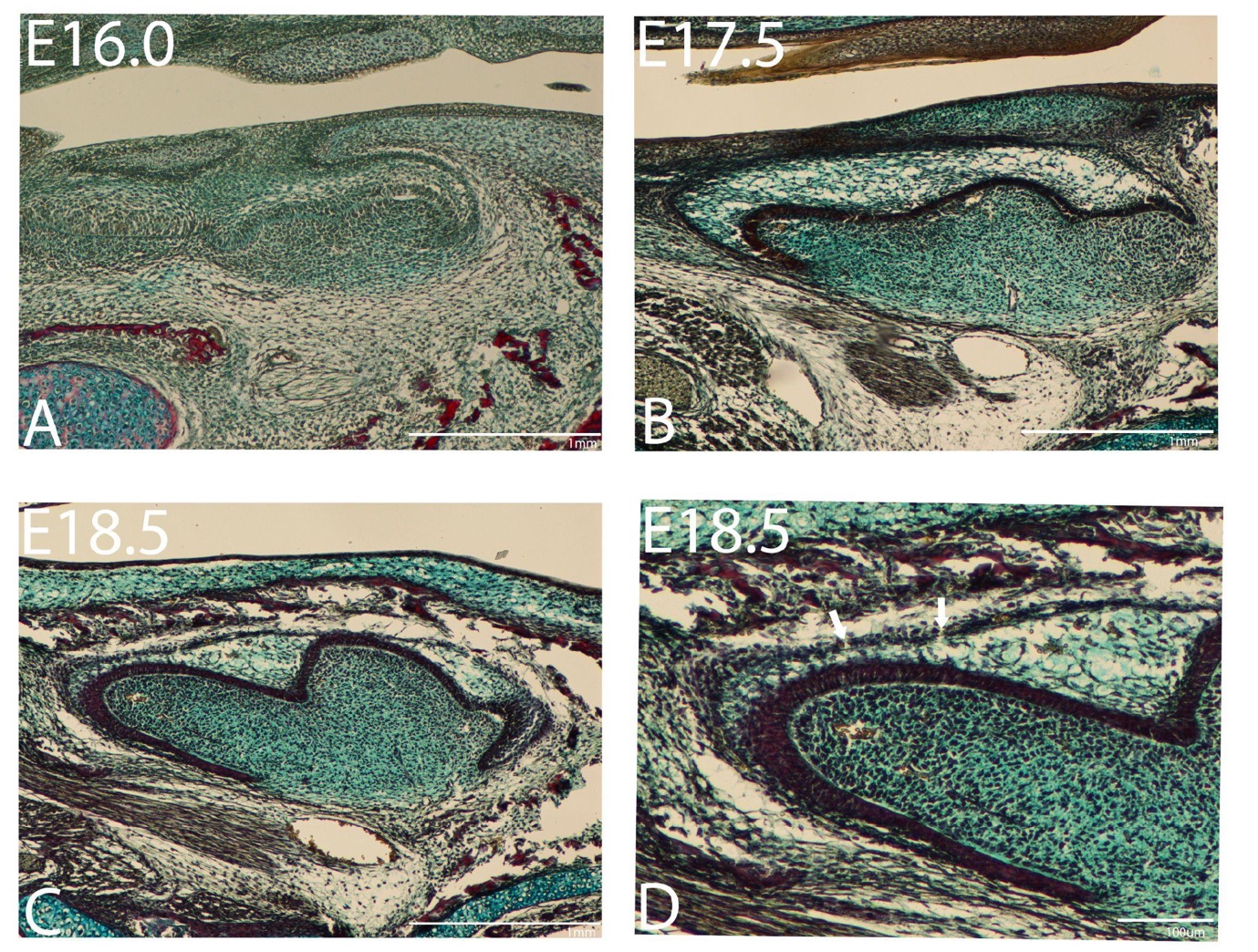


Stages of tooth development, mouse sagittal sections. (A) E16.5, (B) E17.5, (C) E18.5). (D) Magnification of C showing breaks in the OEE (arrows).

Appendix Figure 2: Immuno negative control molar tooth E16.5.

(A) No primary antibody. Blood cells auto-fluoresce in the green channel. (B) CD34 immuno highlighting endothelial cells. (C) Dapi nuclear stain.


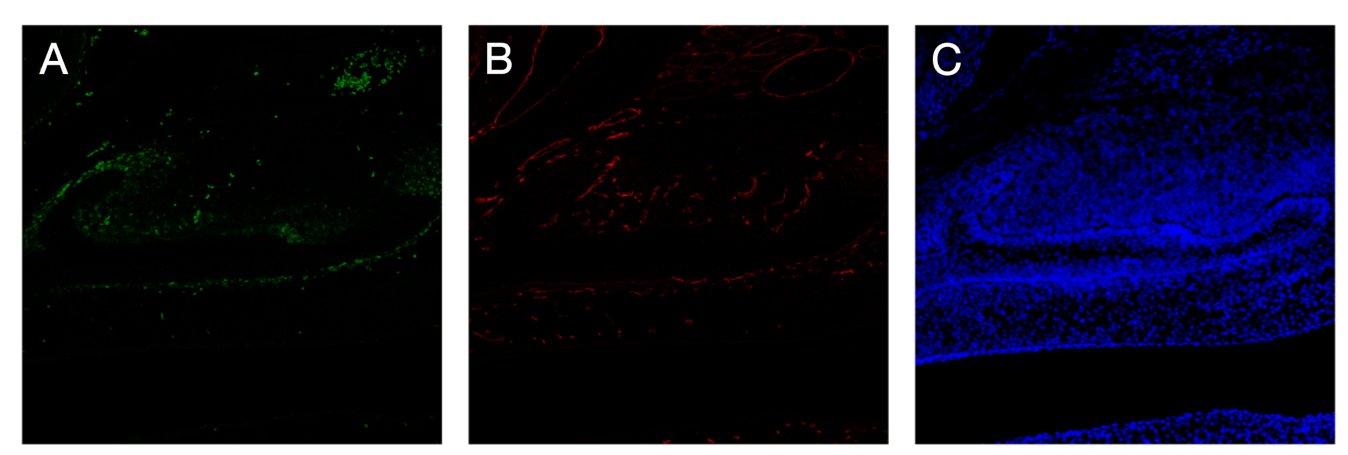


Appendix Figure 3: Measurements of extent of endothelial invasion


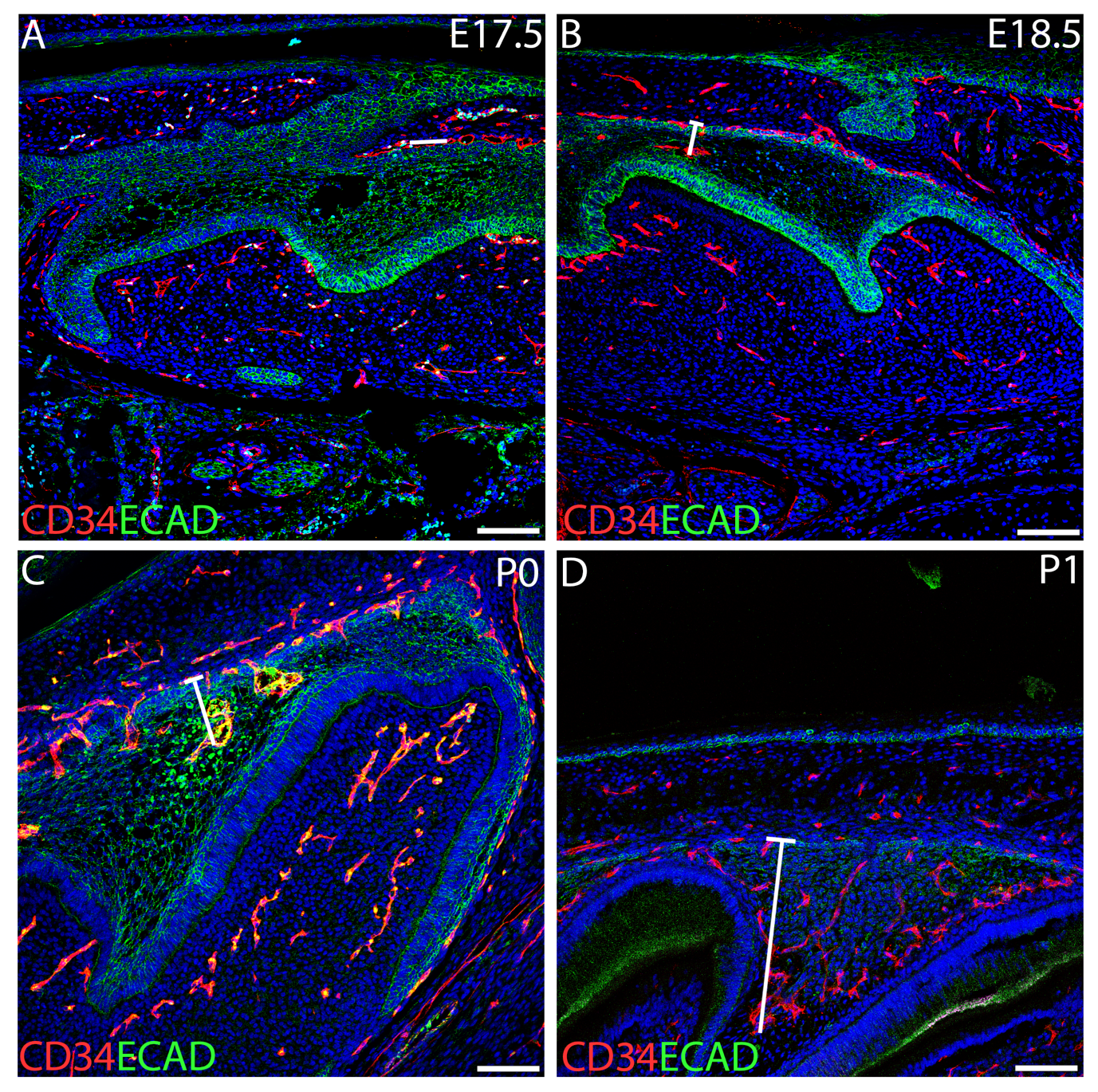


Immunofluorescence for Endothelial cells (CD34) and epithelium (ECAD). To measure the extent of endothelial invasion the furthest endothelial cells from the outer enamel epithelium (OEE) was measured as shown in A-D. Lines placed at 90 degrees to OEE. Points shown in graph Figure 1I.

Appendix Figure 4: Correlation of onset of amelogenin expression and invasion of the vasculature


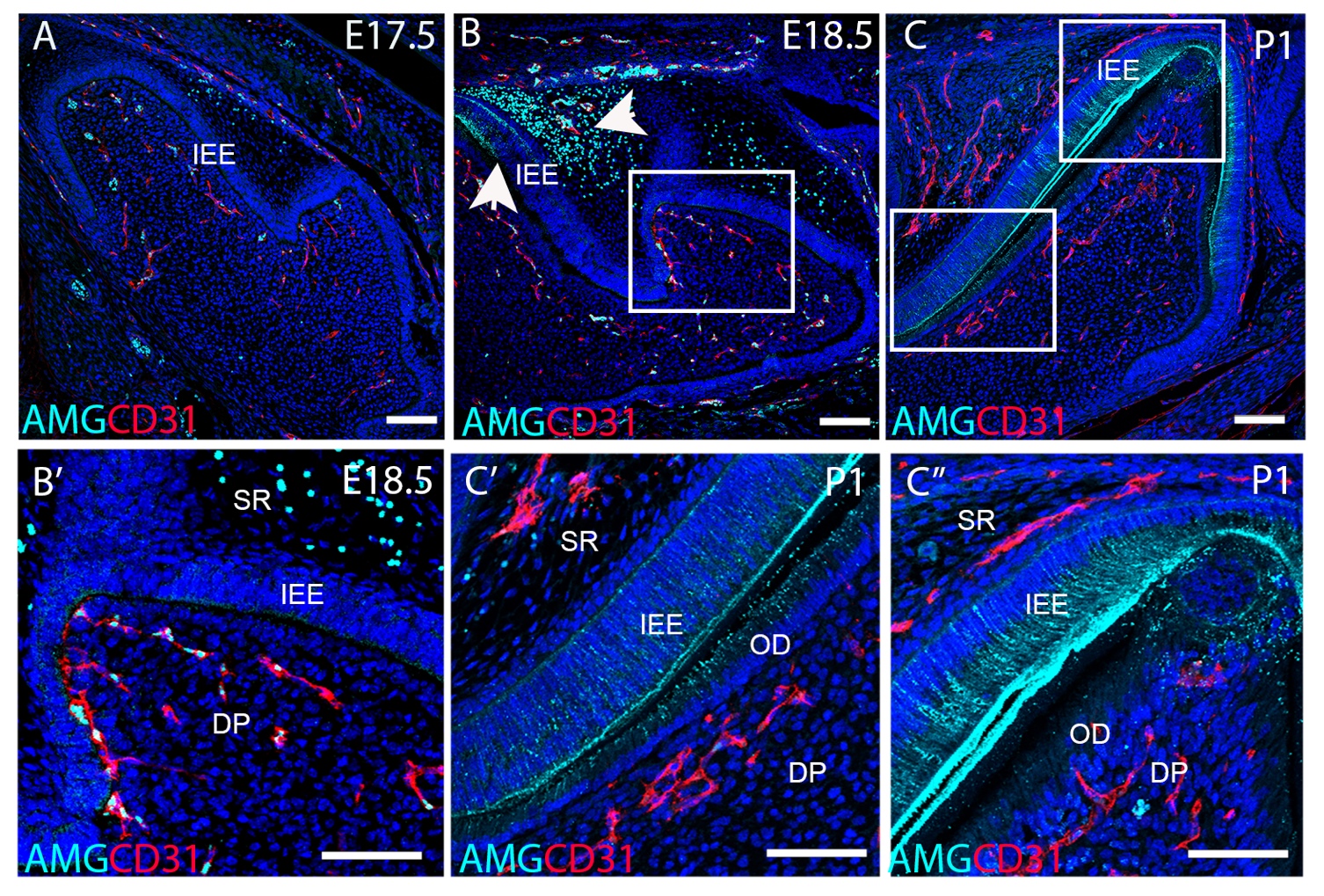


Amelogenin in cyan, CD31 (endothelial cells) in red. (A) E17.5 mouse. No amelogenin in the Inner enamel epithelium (IEE). (B, B’) E18.5 mouse. (B) Arrows points to onset of amelogenin expression at the tip of the cusps, and to extent of blood vessel invasion. Cyan spots in SR are background. (B’) Highpower of box in B. The ameloblasts of the IEE do not have a columnar morphology, and do not express amelogenin (cyan). The endothelial cells are observed in the dental papilla (DP) but have not reached the ameloblasts through the stellate reticulum (SR). (C, C’, C”) P1 mouse. Amelogenin expression has spread towards the apex of the tooth. (C’) High power of lower box in C. Amelogenin is starting to come on and the ameloblasts in the IEE are elongated and close to the endothelial cells in the SR. (C”) High power of upper box in C. Robust expression of amelogenin is observed in the IEE in the region where the endothelial cells are in close contact with the stratum intermedium. OD = odontoblast later. Scale bar in A-C = 100uM. Scale bar in B’,C’,C” = 100um.
